# Surface and bulk modifications of amphibole asbestos in mimicked gamble's solution at acidic PH

**DOI:** 10.1038/s41598-021-93758-9

**Published:** 2021-07-09

**Authors:** Alessandro Pacella, Paolo Ballirano, Marzia Fantauzzi, Antonella Rossi, Elisa Nardi, Giancarlo Capitani, Lorenzo Arrizza, Maria Rita Montereali

**Affiliations:** 1 Dipartimento di Scienze della Terra and Laboratorio Rettorale Fibre e Particolato Inorganico, Sapienza Università di Roma, P.le A. Moro 5, 00185 Rome, Italy; 2 Dipartimento di Scienze Chimiche e Geologiche, INSTM Research Unit, Centro Grandi Strumenti, Università di Cagliari, 09042 Monserrato, Cagliari, Italy; 3 Istituto Superiore per la Protezione e la Ricerca Ambientale (ISPRA), via Vitaliano Brancati 48, 00144 Roma, Italy; 4 Dipartimento di Scienze dell’Ambiente e di Scienze della Terra, Università degli Studi di Milano-Bicocca, Piazza della Scienza 4, 20126 Milano, Italy; 5 Centro di Microscopie, Università degli Studi dell’Aquila, Via Vetoio (Coppito 1, Edificio “Renato Ricamo”), 67100 Coppito, L’Aquila, Italy; 6 ENEA, C.R. Casaccia via Anguillarese 301,S. Maria Di Galeria, 00123 Roma, Italy

**Keywords:** Analytical chemistry, Environmental sciences

## Abstract

This study aimed at investigating the surface modifications occurring on amphibole asbestos (crocidolite and tremolite) during leaching in a mimicked Gamble’s solution at pH of 4.5 and T = 37 °C, from 1 h up to 720 h. Results showed that the fibre dissolution starts with the release of cations prevalently allocated at the various *M*- and (eventually) *A*-sites of the amphibole structure (incongruent dissolution). The amount of released silicon, normalized to fibre surface area, highlighted a leaching faster for the crocidolite sample, about twenty times higher than that of tremolite. Besides, the fast alteration of crocidolite promotes the occurrence of Fe centres in proximity of the fibre surface, or possibly even exposed, particularly in the form of Fe(II), of which the bulk is enriched with respect to the oxidized surface. Conversely, for tremolite fibres the very slow fibre dissolution prevents the underlying cations of the bulk to be exposed on the mineral surface, and the iron oxidation, faster than the leaching process, significantly depletes the surface Fe(II) centres initially present. Results of this work may contribute to unravel possible correlations between surface properties of amphibole asbestos and its long-term toxicity.

## Introduction

Asbestos is a generic commercial term collectively designating six naturally occurring mineral fibres belonging to the serpentine group (chrysotile) and the amphibole super-group (asbestiform varieties of riebeckite, grunerite, anthophyllite, tremolite, and actinolite)^1^, widely recognized to be human carcinogens^2–4^. However, there is still a lack of understanding on the role of the various mineralogical species in modulating the risk associated to asbestos exposure: one part of the scientific and political community assumes that all regulated asbestos fibres are indistinctly classified as potentially toxic substances; the other part promotes the safe use of chrysotile and claims that only amphibole asbestos minerals should be considered carcinogenic^5^. Such hypothesis is based on epidemiological studies showing that low to moderate exposure to chrysotile asbestos has little potential for producing mesothelioma^6,7^, and this is presumably due to its low bio-durability, during phagocytosis, in the intracellular macrophage environment. On the other hand, the bio-solubility of amphibole asbestos is very low (high bio-durability) while amphibole fibres evidence a preferential retention in the lung compared to chrysotile^8–12^. Besides, it was proposed that asbestos tremolite might contribute to the incidence of mesotheliomas and lung cancers even when present as low-level contaminant of chrysotile asbestos^13^.

Recent studies performed by our group on the pathogenic-related surface reactivity of fibrous amphiboles (crocidolite and tremolite) show that the radical production depends on specific surface Fe sites rather than the total Fe content of the minerals^14–16^. In particular, the investigation of the chemical reactivity following sample leaching in oxidative medium buffered at pH 7.4 highlights that both fibrous crocidolite and tremolite samples have sustained radical production, even when mineral surface is highly altered by oxidative leaching^15,16^.

It is also shown that fibres may accumulate iron from available sources in a cell and tissue e.g. haemoglobin and plasma transferrin^17^. Such process of fibre surface coating with Fe-rich phases may represent one of the mechanisms of formation of ferruginous bodies, which has been considered a protective reaction of the host to diminish injury mediated by inhaled fibres, making redox active iron at the fibre surface less available. However, some studies suggest that the coated material may still be cytotoxic, increasing free radical production^18–20^ and inducing the formation of single DNA strand breaks in the presence of low molecular chelator or ascorbate^21^.

It is now clear that the interaction of inhaled fibres with body fluids may alter both the biological environment and the surface properties of the fibres themselves. On this basis, the systematic investigations of possible fibre modifications occurring at physiological pH are very effective to predict how fibres may react with their biological surrounding.

In this work the surface chemical modifications, the iron speciation of amphibole asbestos incubated at 37 °C in a mimicked Gamble’s solution (MGS) at pH 4.5 and the dissolution are investigated. In particular, we focus on crocidolite and tremolite, due to their highly different Fe contents. Fibres were suspended in the leaching solution from 1 to 720 h (one month) and then investigated by a multi-analytical approach. Even being far from mimicking a real cellular environment, such conditions were chosen to promote the dissolution that may occur in vivo in a reasonable experimental time. The morphological investigation of both pristine and reacted fibres was performed by field-emission scanning electron microscopy (FE-SEM), the ion release into the leaching solution was monitored by inductively coupled plasma optical emission spectrometry (ICP-OES); modification of surface chemistry, including Fe(II) and Fe(III) speciation, was investigated by x-ray photoelectron spectroscopy (XPS); structural state of the fibres was studied by both x-ray powder diffraction (XRPD) and high-resolution transmission electron microscopy (HR-TEM).

## Results

### UICC crocidolite

**Figure 1:**
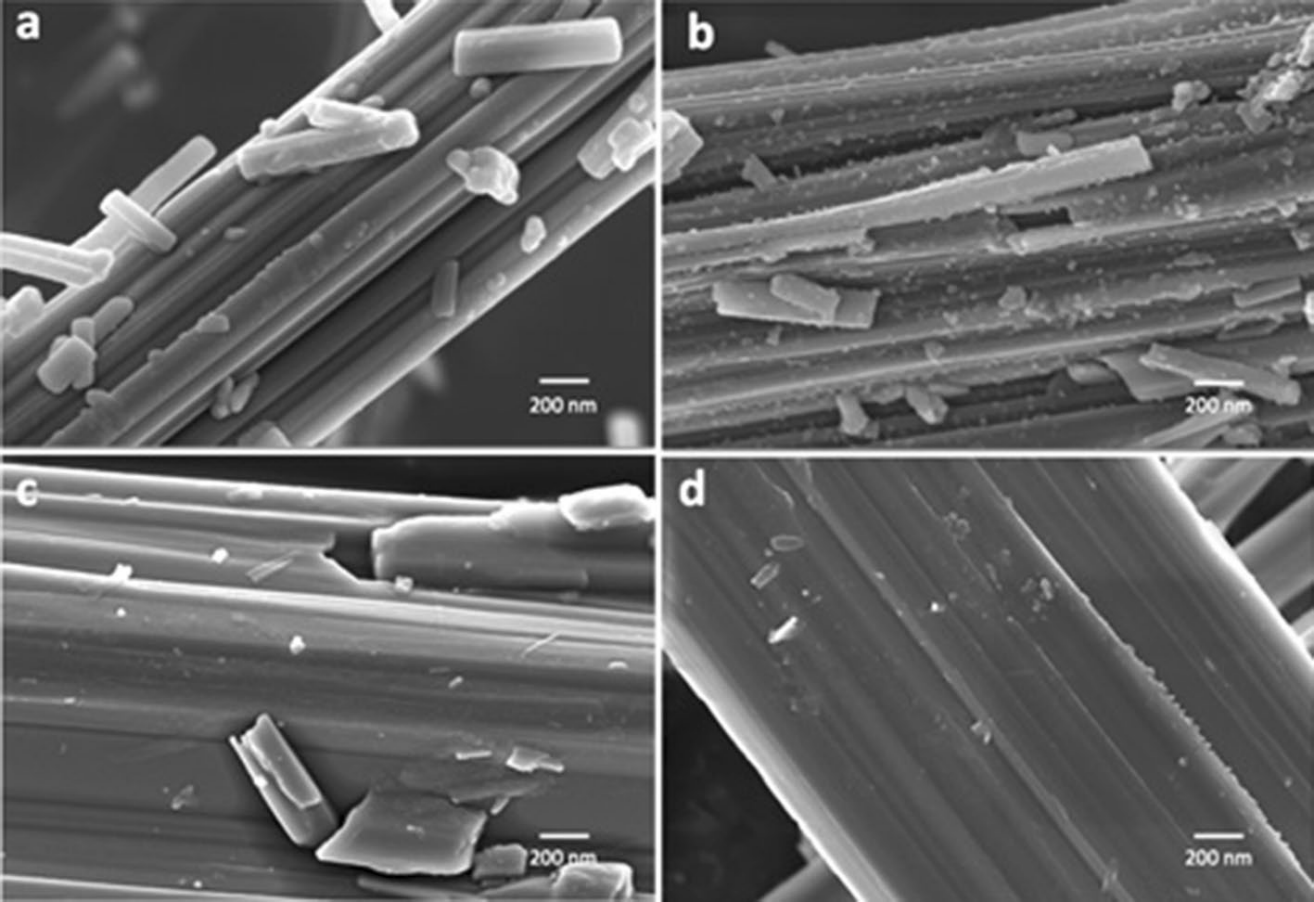
High magnification FE-SEM images of the investigated asbestos samples: (**a**) pristine and (**b**) incubated (sample C-1 M) UICC crocidolite fibres, (**c**) pristine and (**d**) incubated (sample T-1 M) Maryland tremolite fibres. Relative scale bars: 200 nm for (**a**) and (**b**) images, 200 nm for (**c**) and (**d**) images.

Low magnification FE-SEM images indicate that crocidolite fibres are rigid and generally straight and are arranged in bundles characterized by dimensions of ca. 1–3 μm × 50–300 μm (diameter × length). Bundles show split ends and often form felt-like aggregates possibly engulfing the accessory phases (Fig. S1 Supplementary material). At high magnification (Fig. 1a), fibres show an evident kinked appearance and irregular surface apparently testifying the occurrence of an amorphous outer layer possibly due to weathering processes, in agreement with previous HR-TEM investigation^14^. This feature is particularly obvious at the edges of the fibrils that are often saw teeth-like. After immersion in MGS for 720 h (sample C-1 M) the extended effects of the dissolution process, consisting in progressive surface kinking and occurrence of highly corroded and very irregular fibre edges, become apparent (Fig. 1b). In addition, particles of size range from tens up to hundreds of nanometres adhere to the surface of the bundles. SEM–EDS analysis did not disclose any compositional inhomogeneity with respect to the underlying fibre bundle, except in some cases the presence of very minor amount of sulphur.

**Figure 2:**
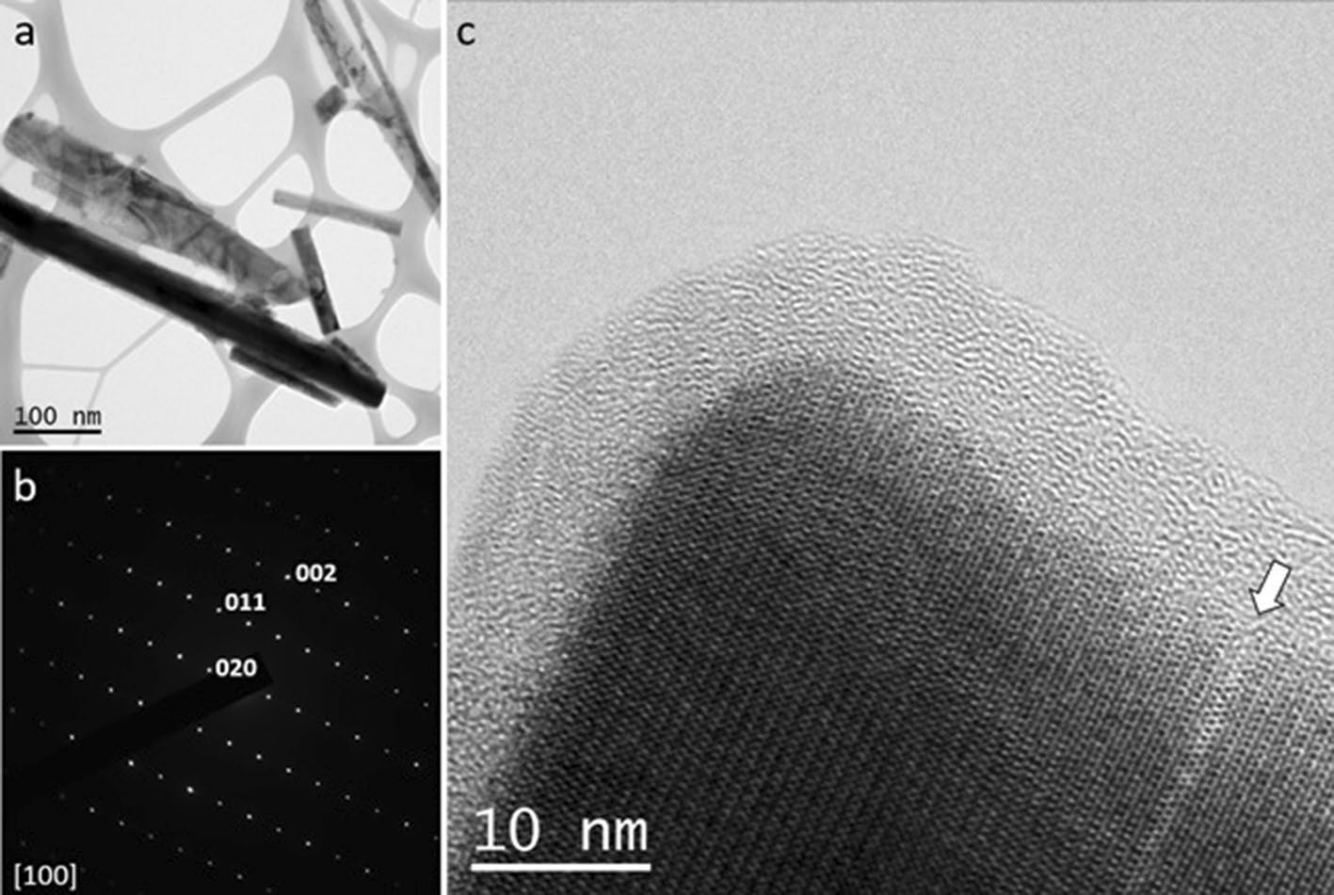
(**a**) Bright field (BF) low magnification image of crocidolite fibres (sample C-1 M) deposited on lacey carbon film. (**b**) Selected area diffraction pattern (SAED) of crocidolite along [100]. (**c**) HR-TEM image of a crocidolite fiber. Note the round corner and the amorphous film of ~ 7–8 nm bordering the crystal suggesting surface dissolution. A single pyroxene lamella one-unit cell wide is visible on the right (arrow).

HR-TEM images of crocidolite, after immersion in MGS for 720 h (sample C-1 M) taken along [100], confirm unequivocally the existence of an amorphous rim of ca. 7–8 nm of thickness (Fig. 2) as well as the occurrence of structural defects. In addition, a few nanometric particles adhering to the surface were identified by the presence of moiré fringes.

Quantitative Phase Analysis (QPA) results of the pristine sample are in very good agreement with reference data of Pacella and co-authors investigations^14^. Prolonged immersion in MGS results in a complete dissolution of carbonates (calcite and siderite) whereas quartz and minnesotaite content remains substantially unaffected (Figure S2). The apparent disappearance of magnetite was, in effect, caused by its removal from MGS by attraction of the magnetic stirrer used to ensure the homogeneity of the suspension. A rough estimate of the amount of this phase in mixture, based on relative intensities, points to content in the 0.1 wt.% range. Moreover, in the C-1 M sample the occurrence of a small reflection at ca. 14° 2θ has been assigned to the 200 reflection of lepidocrocite (γ-FeOOH) and the significant broadening towards lower angles, as compared to that of the pristine sample, of the peak located at ca. 18° 2θ (inset in Figure S2) has been attributed to the occurrence of hydrated sulphates of Mg and Fe (i.e., pentahydrate, butlerite, melanterite). The same broadening has been observed, albeit to a smaller extent, in the T-1 M sample (see below). Accordingly, detailed FE-SEM analysis of nanoparticles lying at the surface of the crocidolite fibres detected in some cases tiny amount of sulphur, suggesting therefore possible precipitation of such sulphate phases.

Rietveld refinement results showed marginal modifications of riebeckite cell parameters and volume suggesting the occurrence of only very minor changes at the bulk level (Table S1). Variation of bond distances, aggregate size of the constituent cations < r^M^ > (Table S2), and cation sites scattering (Table S3) are small and confined to ± 3σ. As a consequence, the cation partition, carried out following the procedure of Vignaroli and co-authors^22^, is very similar in both samples (Table S4), possibly suggesting a small increase of the Fe(II)/Fe(III) ratio from ca. 1.6 to ca. 1.8 mainly caused by the increase of the Fe(II) content at *M*(1) at the expense of Fe(III).

**Figure 3:**
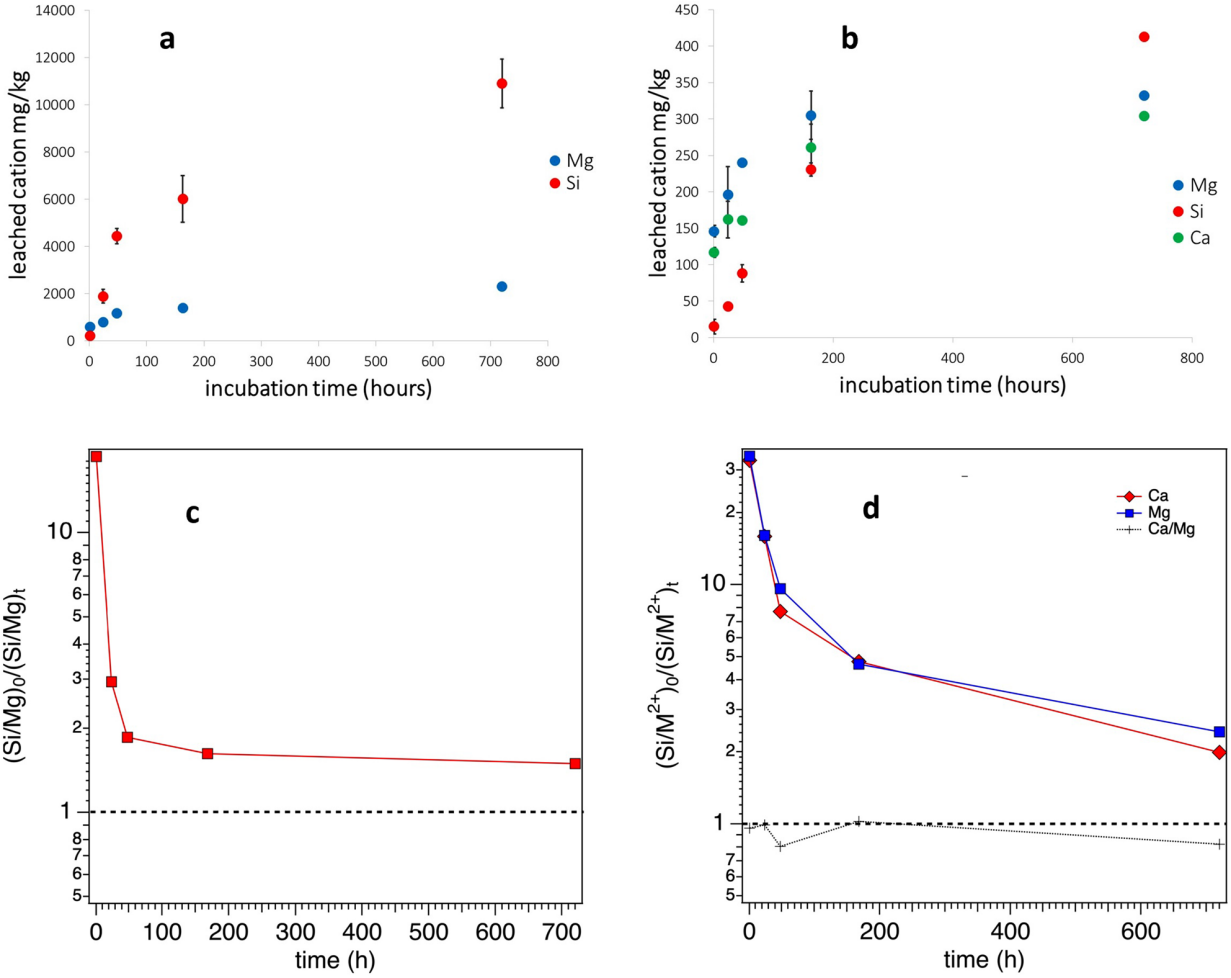
Dissolution of the investigated fibrous samples in the simplified Gamble’s solution at pH 4.5 in the range 0–720 h: (**a**) UICC crocidolite; (**b**) Maryland tremolite. Variation of the Si/M^2+^ ratio (M^2+^  = Mg, Ca) of the released cations at each time of sampling t [(Si/M^2+^)_t_] as compared to that arising from chemical analyses of the pristine sample at the start of the dissolution process [(Si/M^2+^)_0_]: (**c**) UICC crocidolite and d) Maryland tremolite.

Results of ICP-OES analyses after UICC crocidolite fibres incubation in the MGS are reported in Fig. 3a and Table S5. Ca release mainly occurs in the early steps of sample incubation (10,814 mg/kg). It is worth noting that SEM/EDS analyses did not detect the presence of Ca in the UICC crocidolite fibres^15^, and therefore the observed release was mainly attributed, following QPA, to the dissolution of small amounts of calcite (calcium carbonate: CaCO_3_) occurring in the pristine sample (ca. 1.1 wt.%). On this basis, the significantly reduced Ca release observed after 168 h of incubation time is most likely due to different amounts of calcite at the start of the experiments in the various tubes caused by some degree of inhomogeneity of the hand sample. Similarly, the observed release of Fe is due to the dissolution of siderite (iron carbonate: FeCO_3_), in agreement with the results of QPA obtained by Rietveld refinements. Notably, the complete dissolution after 168 h of about 1.3 wt.% of siderite present in the pristine sample should produce the release of about 6000 ppm of Fe into the solution. The presence of only about 2500 ppm indicates the possible precipitation of Fe, under the form of Fe-bearing phases. This hypothesis is further confirmed by the occurrence of lepidocrocite (γ-FeOOH) and possibly Fe-bearing hydrated sulphates in the C-1 M sample, as revealed by XRPD data (Table 1). Moreover, Fe release from magnetite was ruled out since this phase is not soluble at our experimental conditions, as further confirmed by dissolution experiments performed on a pure Fe_3_O_4_ sample.

**Table 1:** Quantitative Phase Analysis of the UICC crocidolite samples. Serpentine-like and melanterite were not quantified (see text for explanation).

Phases	Pristine	C-1 M
Crocidolite	93.50 (13)	97.04 (11)
Magnetite	1.86 (4)	−
Quartz	1.58 (3)	1.90 (3)
Calcite	1.15 (8)	−
Siderite	1.32 (9)	−
Minnesotaite	0.60 (4)	0.62 (4)
Lepidocrocite	−	0.44 (10)
Serpentine-like	+	+
Mg,Fe hydrated sulphates	–	+

Both Si and Mg release increase with incubation time (720 h), ranging from 221 mg/kg up to 10,900 mg/kg, and from 586 mg/kg up to 2307 mg/kg, respectively (Fig. 3a, Table S5). For both cations, the observed leaching trend reveals a higher fibre dissolution rate in the first 48 h of incubation time.

The XPS survey spectra of crocidolite samples after suspension in MGS are reported Figure S3 and show the presence of Si, O, Fe, Mg and Na together with some C due to the organic contamination layer. Notably, no Cl and S signals that might be due to the incubating solution were detected on the fibre surface. The binding energy of the main photoelectron lines are listed in Table S6. The high-resolution spectra were processed to obtain information on the chemical state of the elements.

**Figure 4:**
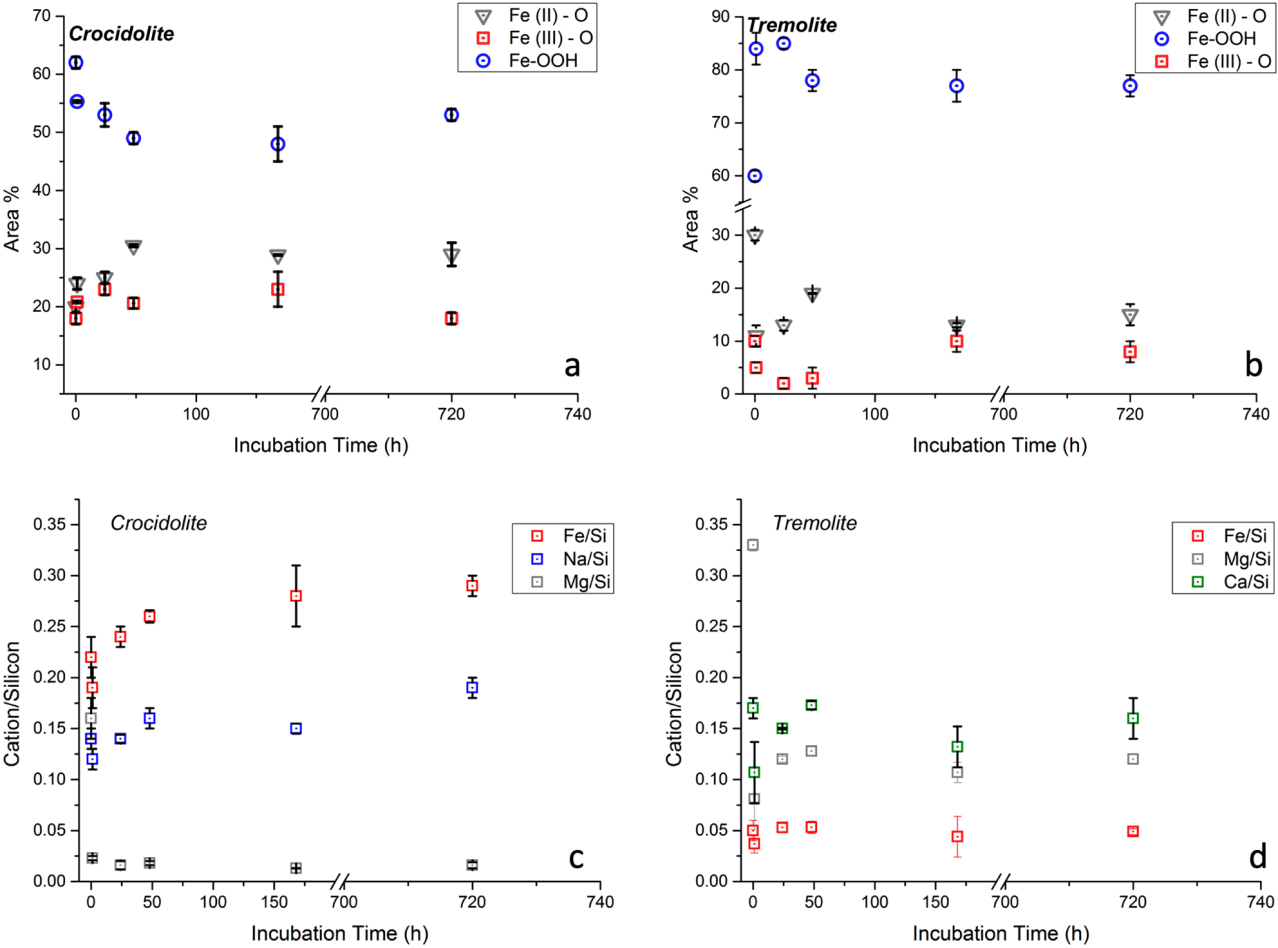
Relative intensities of the Fe 2p_3/2_ components (area%): (**a**) UICC crocidolite and (**b**) Maryland tremolite. Cations/silicon ratios vs incubation time: (**c**) UICC crocidolite and (**d**) Maryland tremolite.

Si 2p peaks were fitted with a doublet due to spin orbit coupling. The energy separation between the 2p_3/2_ and 2p_1/2_ components and their area ratio were constrained to 0.8 and 2:1, respectively. The binding energy of Si 2p_3/2_ (Table S6) agrees with those reported in our previous investigations^23,24^ for both crocidolite pristine and incubated in phosphate buffer solution samples. Oxygen O 1 s peak resulted to be multicomponent with a signal due to oxygen in oxides in the range 530.0–530.2 eV, the component due to non-bridging oxygen in silicates and -OH are found at about 531.2 eV and the component assigned to bridging oxygen is at 532.1 eV^23–25^. The surface quantitative composition of the incubated fibres is reported in Table S7, together with the composition of the pristine crocidolite fibres. It has to be pointed out that, owing to their very low amount, the contribution of accessory phases to the XPS measurements is negligible. Notably, the surface composition of the pristine fibres shows an enrichment in Si and Mg coupled with a significant depletion in Fe, suggesting the occurrence of weathering processes of the fibres, as already mentioned in the Materials and Method section. The Fe 2p_3/2_ high-resolution spectra of the samples are shown in Figure S4. Following Fantauzzi and co-authors^24^, the signals were resolved in three components assigned to Fe(II) bound to oxygen with its satellite, Fe(III) bound to oxygen and FeOOH. The percentage of each component is shown in Fig. 4a and is reported in Table S8. In particular, the intensity of the Fe(II)-O signal increases almost regularly from 20% of the total peak area in the pristine sample up to 30% after 48 h of dissolution and then it remains unchanged; the Fe(III)-O signal is roughly constant (values at about 20%) whereas the Fe(III)-OOH signal decreases from 62 to 49% in the first 48 h and then it remains constant (values about 50%). This behaviour is consistent with the small increase of the Fe(II)/Fe(III) ratio observed within the bulk from Rietveld refinements.

### Maryland tremolite

FE-SEM images show that tremolite fibres are more inhomogeneous than crocidolite from the dimensional point of view as they occur as both small and large bundles whose diameter span from ca. 1 to 10 μm. Small fibrils of ca. 50 μm in length are also present (Fig S1). At high magnification, tremolite shares essentially the same pre-existing alteration features of crocidolite albeit to a much more limited extent (Fig. 1c) such as indicating an early stage of a similar process of alteration shown by the other amphibolic fibres.

After immersion in MGS for 720 h (sample T-1 M), tremolite fibres do not show significant modifications as compared to the pristine sample, albeit the teeth-like aspect of the fibre edges is more evident (Fig. 1d). In addition, nanoparticles lying at the surface of the bundles, which were present in crocidolite, are now rare. However, a few molds at the surface of some bundles testify the ongoing similar process. All those observations clearly point out to a less marked surface alteration for tremolite than crocidolite.

**Figure 5:**
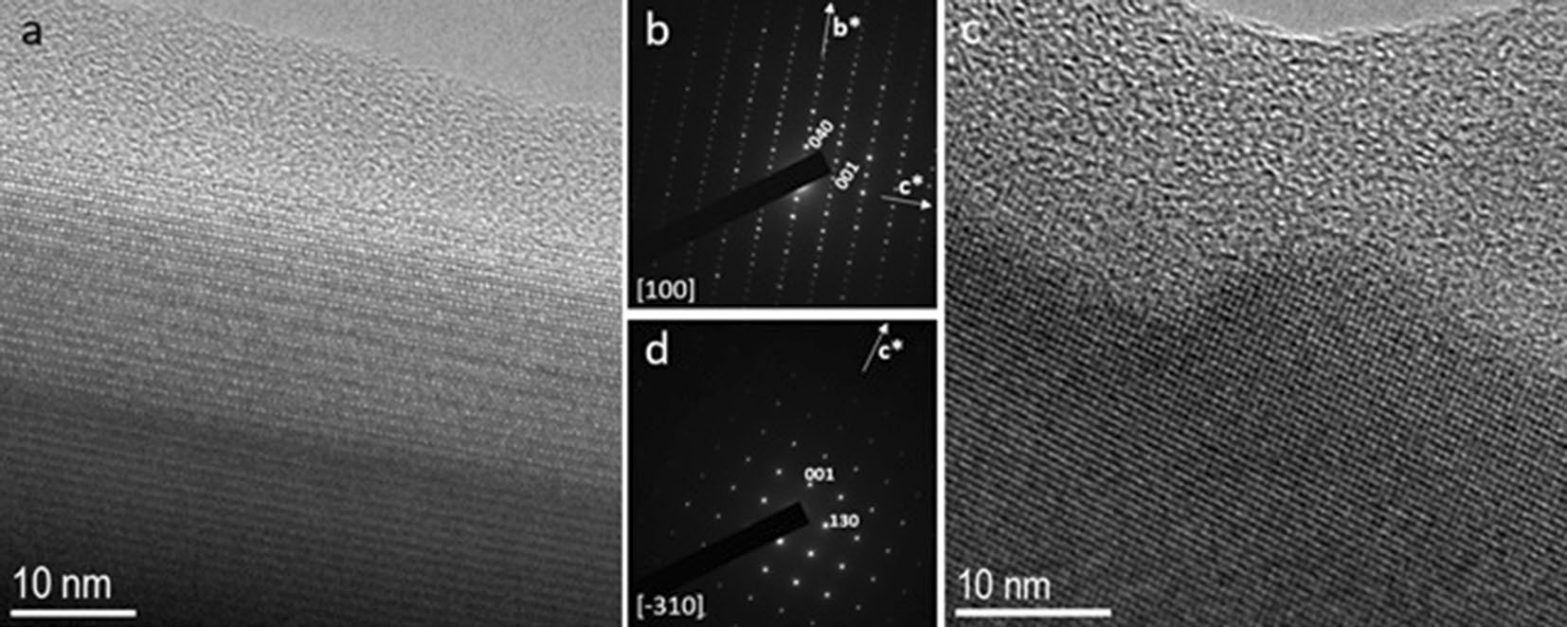
(**a**) HR-TEM image of tremolite (sample T-1 M) taken along [100] showing an amorphous rim of ~ 10–11 nm. (**b**) related SAED pattern. The apparent violation of the C-centering (*h* + *k* = 2n) is probably due to (100) pseudo-merohedral twinning. (**c**) HR-TEM image taken along [$\stackrel{-}{3}10$] and related SAED pattern (**d**). Note the much larger amorphous rim (~ 20 nm).

HR-TEM images of sample T-1 M, taken along the same orientation than crocidolite, namely [100], show an amorphous rim on the order of 10–11 nm (Fig. 5), which is approximately comparable with the thickness observed in the pristine sample. HR images taken along less dense directions in terms of atomic potential (e.g. $\left[\stackrel{-}{3}10\right]$) may show larger amorphous rims (Fig. 5). It is not clear if this is due to dissolution rates varying with the crystal direction or if it is a consequence of the image formation process: less dense directions are more difficult to be imaged in HR mode, therefore resulting aperiodic to a larger extent.

Miscellaneous data of the refinements of the samples of tremolite from Maryland are reported in Table S9. Representation of the experimental data on a logarithmic scale (Figure S5) shows, as in the case of UICC crocidolite samples, a very small peak at ca. 12° 2θ assigned to the strong 002 reflection of serpentine (possibly at the 0.1 wt.% level). However, in this case the peak disappears upon incubation, indicating the complete dissolution of the phyllosilicate. As in the case of C-1 M, the peak located at ca. 18° 2θ of the T-1 M sample is characterized by relevant broadening towards lower angles (inset in Figure S5). It is worth noting that, according to the absence of iron-bearing accessory phases, as compared to the UICC crocidolite sample, no reflections attributable to lepidocrocite were observed in the T-1 M sample. Modifications of cell parameters and volume are very small and point out to a minor contraction potentially attributable to minor Fe(II) oxidation^26^.

Variation of bond distances, aggregate sizes of the constituent cations < r^M^ > (Table S10), and cation sites scattering (Table S11) are confined to ± 3σ indicating no effect of the dissolution process at the bulk level. The cation partition of the pristine and incubated samples are very similar (Table S12) suggesting a moderate decrease of the Fe(II)/Fe(III) ratio from ca. 3.4 to ca. 2.0 mainly caused by partial oxidation occurring at *M*(2). The reduction of the Fe(II)/Fe(III) ratio is supported by the observed small cell parameters contraction and should be counterbalanced by (very minor) de-protonation in order to maintain charges neutrality.

Results of ICP-OES analyses following the tremolite sample incubation in MGS are reported in Fig. 3b and Table S13. In particular, the release of Si progressively increases with sample incubation time, ranging from 15 mg/kg after 1 h up to 543 ppm after 720 h. In addition, Mg and Ca release shows an increasing trend during the first 168 h of incubation (values ranging from 146 mg/kg to 305 mg/kg and from 117 mg/kg to 261 mg/kg, respectively), and then becoming constant until the end of the experiment (Fig. 3b and Table S13). Notably, the release of cations, due to the leaching, revealed that a faster dissolution is occurring in the first 168 h of incubation. Moreover, Fe release from the sample was not directly observed for any dissolution time (for the experimental conditions adopted here the detection limit for Fe was 50 µg/kg).

The survey spectra of tremolite samples following incubation in the MGS solution show the presence of Si, O, Fe, Mg, Ca and small amounts of Na together with some C due to the organic contamination layer (Figure S6). The binding energy values of the photoelectron lines are reported in Table S14. Similarly, to crocidolite, Fe 2p_3/2_ and O1s signals detected for tremolite were multicomponent. The surface quantitative composition of both pristine and incubated fibres is reported in Table S15. It must be pointed out that the contribution in sample XPS measurements of the impurity (ca. 0.1 wt.% of serpentine) is negligible. As observed for UICC crocidolite, the surface composition of the pristine sample shows differences with respect to that of the bulk, being enriched in Si and depleted both in Mg and Ca, suggesting that the fibres underwent weathering processes. The Fe 2p_3/2_ spectra of the incubated samples are shown in Figure S7. The percentage of each component of Fe 2p_3/2_ is provided in Fig. 4b and is reported in Table S16. In particular, the intensity of both the Fe(II)-O and Fe(III)-O components sharply decrease from 30 to 11% of and from 10 to 5% of the total Fe peak area in the first hour of incubation, respectively. Conversely, during the same incubation time, the intensity of the Fe(III)-OOH components increases from 60 to 84% of the total Fe peak area. For longer incubation times, the intensity of the Fe(II)-O component is almost invariant, except for the value of 19% observed after 48 h of incubation time. Moreover, the Fe(III)-O component is restored at values comparable to that of the pristine sample (10%) after 168 h and then is constant, whereas the Fe(III)-OOH slightly decreases in the range of incubation time 1–48 h and then it remains almost constant.

## Discussion

The dissolution of UICC crocidolite fibres leads to the release of Si, Mg, Ca, and Fe from the sample (Fig. 3a and Table S5). Notably, there is not contribution to Si leaching from the accessory silicate phases present in the hand sample since QPA analysis revealed that neither quartz nor minnesotaite dissolution occurred. On the contrary, the observed release of Ca and Fe was attributed to the dissolution of small amount of calcite and siderite, respectively; these two minerals occur in the hand sample and the results are in agreement with the well-known solubility of carbonate minerals in acidic solutions. Therefore, the estimated maximum amount of released Si and Mg from the fibres is about 2% and 7% of the total content, respectively.

Figure 3c reports the variation of the Si/Mg ratio of the released cations (based on nanomoles) at each time of sampling [(Si/Mg)_t_] as compared to that arising from chemical analyses of the surface of the UICC crocidolite pristine sample at the beginning of the dissolution process [(Si/Mg)_0_]. As can be seen at each time of the dissolution process, Mg is preferentially released compared to Si, due to differences in Madelung site energy^27,28^. Moreover, the (Si/Mg)_0_/(Si/Mg)_t_ ratio decreases with a very fast trend, by an order of magnitude, in the first 48 h and then very slowly, highlighting that the dissolution of cations is still incongruent (i.e. non-stoichiometric cation release with respect to the surface composition of the pristine sample), even if to a less extent, for longer incubation times. These results are in good agreement with those obtained by XPS on the fibre surface, where a decrease of Mg/Si ratio coupled with an increase of the Fe/Si ratio upon incubation time is observed (Fig. 4c). Similarly, the increase of the Na/Si ratio upon incubation time (Fig. 4c) indicates no Na solubility. Notably, the cations hosted at the *M*(4) site of the amphibole structure (Na^+^ and Ca^2+^), are expected to be rapidly leached out during amphibole dissolution, on the basis of Madelung site energy^23,25,27,29^. Possible explanation of our result is that the high Na concentration in MGS hinders Na leaching (i.e. common ion effect).

Pristine UICC crocidolite fibres show that the bulk is enriched in Fe(II) with respect to the surface (Fe(II)/Fe_tot_ ratios are 52% for the bulk and 20% for the surface). Moreover, Fe(III) is mainly present under the form of FeOOH (62% of the total surface Fe content). On this basis, the increase of the Fe(II) content at the fibre surface (from ca. 20% to ca. 30% of the total Fe content, see Fig. 4a and Table S8) and the decrease of the FeOOH component observed in the first 48 h of incubation is related to the dissolution process that eroding the outer layer promotes the bulk Fe sites near the fibre surface. Notably, the increase of Fe(II) reaches a maximum in the first 48 h, where the dissolution process is particularly vigorous (Fig. 3a). For longer incubation time the two processes likely approach equilibrium, as the Fe(II) content on the surface remains almost constant, therefore suggesting that, in the early steps, the dissolution is faster than the oxidation (Fig. 4a and Table S8).

Results from the dissolution process of the Maryland tremolite sample indicate the small release of Si, Mg and Ca from the mineral bulk (Fig. 3b and Table S13) well below 0.5% (0.09, 0.12, and 0.28%, respectively). These findings agree with previous works that reported a more rapid dissolution for the iron-rich minerals with respect to their isostructural, iron-free analogues^29,30^. Notably, the release of Fe from the fibres was not directly observed for any dissolution time, since in the experimental conditions used (pH of 4.5 and in the presence of air) Fe cannot be present in solution, even at extremely low concentration.

Figure 3d shows the variation of the Si/M^2+^ ratio (M^2+^  = Mg, Ca) of the released cations at each time of sampling [(Si/M^2+^)_t_] as compared to that arising from chemical analyses of the surface of the Maryland tremolite pristine sample at the beginning of the dissolution process [(Si/M^2+^)_0_]. Similarly to UICC crocidolite, at each point of the dissolution process, Ca and Mg cations are released at a higher rate than Si, as expected following their Madelung site energy^27,28^. The (Si/M^2+^)_0_/(Si/M^2+^)_t_ ratios decreases with the same trend observed in UICC crocidolite indicating that, for both amphibole asbestos, the cation dissolution is incongruent in the whole duration of the experiment. However, a few differences are observed. As far as tremolite is referred to, the Ca/Mg release ratio is found to be in the range 0.8–1 within the analysed time span. This value is more than the double of the stoichiometric one equal to 0.4. Therefore, in the same time span, the release of cations from the *M*(4) site is significantly enhanced as compared to *M*(1,2,3) sites. This behaviour is not replicated by crocidolite, possibly due to common ion effect (see above). Moreover, the (Si/Mg)_0_/(Si/Mg)_t_ ratio approaches the value of one significantly faster than in crocidolite (1.49 vs 2.42 after 720 h).

It must be pointed out that the amount of Si and Mg related to tremolite dissolution might be slightly overestimated since it incorporates the cation release due to the serpentine dissolution. This amount in the sample was not quantifiable by the Rietveld method due to the small intensities associated to this phase and to the interference with the peaks belonging to other mineral phases occurring in the mixture. In addition, the amount of Si and Mg released from serpentine becomes even more difficult to quantify if we would consider that the structure amorphization, following partial dissolution of the mineral, might cause the disappearance of the peaks from the XRPD pattern^31^. Nevertheless, ICP results are in good agreement with those obtained by XPS on the fibre surface, where a significant decrease of the Mg/Si and Ca/Si ratios in the first hour of incubation is observed (Fig. 4d), when the dissolution process is particularly strong (Fig. 3b and Table S13). Notably, the presence of small amounts of Na on the surface of the incubated fibres (Table S15) is likely due to the incorporation of Na cations from the solution into surface sites of the amphibole structure via ion exchange process with Ca ions. Tremolite fibres have the bulk enriched much more in Fe(II) with respect to the surface (Fe(II)/Fe_tot_ ratios are 85% and 30%, respectively) and Fe(III) is present on the surface mainly as FeOOH (FeOOH accounts for 60% of the total surface Fe content). The intensity of the Fe(II)-O component obtained by XPS at the fibre surface strongly decreases in the first hour of incubation (from ca. 30% to ca. 11% of the total Fe) and is counterbalanced by the increase of the Fe(III)-OOH component (from ca. 60% to ca. 84% of the total Fe), suggesting that in this range of time for Maryland tremolite the Fe oxidation is faster than the fibre dissolution (Fig. 4b and Table S16). Notably, the observed behaviour is opposite to that observed for UICC crocidolite, in agreement with our previous findings^23,25^ for dissolution experiments on the same samples at neutral pH and oxidizing conditions. Moreover, for tremolite most of the surface iron is immediately turned into FeOOH (Fig. 4b and Table S15). In a previous work^25^ we proposed that this observation might be related to the slow dissolution of the fibres, which in turn should be affected by their low surface area (2.75 m^2^g^−1^ for Maryland tremolite and 8.66 m^2^g^−1^ for UICC crocidolite).

Since it is well known from literature that body fluids are continually replenished^8^, in order to compare the fibre biodurability of the investigated asbestos samples, the dissolution rates were quantified using Si release in the unsaturated region (0–48 h and 0–168 h, for UICC crocidolite and Maryland tremolite, respectively). The comparison of Si release rates based on equivalent surface area is ca. 19 times larger for UICC crocidolite than for tremolite [dSi/dt = 0.007 µmol × h^−1^ × m^−2^ (R^2^ = 0.99) and dSi/dt = 0.0004 µmol × h^−1^ × m^-2^ (R^2^ = 0.99), respectively]. Notably, the Si release rate obtained for Maryland tremolite (dSi/dt = 0.025 µM × h^-1^) well agrees with that reported by Oze and Salt^8^ for tremolite incubation in modified Gamble’s solution at pH 7.4 (dSi/dt from 0.015 µM × h^−1^), using the same solid/liquid ratio (0.5 mg/mL).

## Conclusions

In this work the mechanism of tremolite and crocidolite fibres leaching was investigated in acidic medium (pH of ca. 4.5) in the presence of air. The fibre dissolution starts to affect the first surface layers that incongruently release the cations prevalently allocated at the various *M*- (and eventually *A*-) sites of the amphibole structure. Disregarding the very small contamination of the Maryland sample due to accessory serpentine and considering the Si release based on equivalent surface area, the comparative study of the two amphibole asbestos samples highlighted that crocidolite is less biodurable than tremolite, being its Si leached roughly twenty times faster. Besides, the more intense dissolution and surface alteration of crocidolite promotes the occurrence of iron centres in proximity of the fibre surface, particularly under the form of Fe(II), of which the bulk is enriched with respect to the oxidized surface. The removal of the first atomic layers during dissolution leads these bulk iron centres to be even exposed on the mineral surface. Moreover, the erosion of the silicate framework may induce coordinative unsaturation of these sites, making them able to react with the surrounding environment. Conversely, for tremolite the markedly slower dissolution of the silicate framework of the fibres prevents the underlying cations of the bulk to be exposed on the mineral surface. Furthermore, iron oxidation, taking place at faster rate than the leaching process, significantly depletes the surface Fe(II) centres initially present.

It must be noted that biodurability is assumed to play a primary role in the long-term toxicity and pathogenicity of fibres deposited in the body^31^. The different biodurability exhibited by crocidolite and tremolite may be responsible for possible distinct behaviour of the two fibres in vivo. On this basis, a comparison of their toxic potential should be done. Besides, considering that asbestos reactivity is strictly related to fibre surface properties, the deep knowledge of the mineral surface modifications occurring at physiological pHs represents a fundamental contribution to unravel possible correlations between physical chemical features of amphibole asbestos and its toxicity. It should be pointed out that both samples underwent an oxidative leaching process in the mimicked lung fluid at acidic pH almost superimposable to that observed in the phosphate buffered solution at neutral pH. However, in this latter case, the large concentration of phosphate within solution (0.5 M, ca. three order of magnitude than that in lung fluids) led to the formation of a surface coating of the fibres with Fe-phosphate nanoparticles, clearly visible by TEM especially for crocidolite. Besides, this coating was proved to modulate fibre chemical reactivity^16^.

## Materials and methods

### Materials

A crocidolite sample from Koegas Mine, South Africa, supplied by the Union International for Cancer Control (UICC), and a fibrous tremolite sample coming from the ophiolitic complex of Montgomery County, Maryland (USA) are investigated in this work. The detailed crystal chemical and structural characterization of both samples was carried out by Pacella and co-authors^32,33^. In particular, the retrieved chemical formula of UICC crocidolite ^A^Na_0.03_^B^Na_2.00_^C^(Fe^2+^_2.21_Fe^3+^_2.04_Mg_0.75_)_Σ5.00_^ T^[Si_7.95_Al_0.02_]_Σ7.97_O_22_^O3^(OH)_2_ was found to be reasonably close to that of the end-member riebeckite Na_2_(Fe^2+^_3_Fe^3+^_2_)Si_8_O_22_(OH)_2_. Structural data pointed out that both Fe(II) and Fe(III) are distributed over the *M*(1,2,3) sites exhibiting the following site-specific occupation preference: *M*(3) > *M*(1) >  > *M*(2) and *M*(2) >  > *M*(1) > *M*(3) for Fe^2+^ and Fe^3+^, respectively.

Quantitative phase analysis (QPA), carried out by the Rietveld method, showed the presence of about 6 wt.% of accessory phases, such as magnetite (1.9 wt.%), quartz (1.6 wt.%), calcite (1.1 wt.%), siderite (1.3 w.t%), and minnesotaite (0.6 wt.%), an iron-bearing phyllosilicate of formula Fe^2+^_3_Si_4_O_10_(OH)_2_ showing minor Mg → Fe(II) substitution.

Tremolite fibres from Maryland have the chemical formula ^B^(Ca_2.00_Mn_0.02_Na_0.01_)_Σ2.03_^C^(Mg_4.48_Fe^2+^_0.44_Fe^3+^_0.08_)_Σ5.00_^ T^[Si_7.95_Al_0.02_]_Σ7.97_O_22_^O3^[(OH)_1.98_F_0.01_].

In this case, Fe(II) was found to be equally distributed over *M*(1), *M*(2) and *M*(3) sites of the octahedral layer, whereas Fe(III) was found only at *M*(2) site.

Both samples show an amorphous rim of various thicknesses, ranging from 5 to 15 nm, as observed by HR-TEM imaging^14^. It is worth noting that the occurrence of a surface layer, which resulted to be Si-rich and amorphous, was consistently observed in many regulated asbestos amphiboles, UICC crocidolite included^34^. Such feature, at least in the case of the analysed samples, may be related to the occurrence of weathering processes reported in some of the various crocidolite-bearing reefs at Koegas-Westerberg^35^ and to the geological conditions required for slip-fibre growth, leading to the shear-stress environment found at Montgomery County^36,37^.

The surface area of the two samples resulted significantly different: 8.66 mg^−1^ for UICC crocidolite^23^ and 2.75 m^2^g^−1^ for Maryland tremolite^15^.

### Dissolution experiments

The leaching solution was made using “ultrapure” deionised water (18.2 MΩ cm at 25 °C) obtained from a MilliQ Element system (Millipore, France) and the following reagents and materials: NaCl RPE-ACS (Carlo Erba Reagents, S.A.S., Milano, Italy), Na_2_SO_4_ • 10H_2_O (Carlo Erba Reagents, S.A.S., Milano, Italy), 67–69% HNO_3_ traceSelect™ Ultra for ultra trace analysis (Honeywell, Fluka, Canada), ultrapure 37% HCl Ultrex (J.T. Baker, Canada). Polypropylene Falcon Tubes (BD Falcon™, Corning, Mexico) were used during sample handling, syringes (BD Plastipack™, Spain) and 0.22 µm GSWP nitrocellulose membrane filters (Millex-HA, Millipore, Ireland) were adopted for sample filtration. pH measurements were performed using a portable 250A Orion pH Instrument (Orion Research Inc., Boston, USA) equipped with an Orion gel-filled combination semi-microelectrode (Thermo Electron Corp., Cambridge, England) for pH measurements.

Dissolution experiments were carried out in a MGS based on the formulation reported in the work by Rozalen and co-authors^38^ (NaCl 112.3 mmolL^-1^ and Na_2_SO_4_ 0.556 mmolL^-1^). In particular, saline solutions have the same molar composition of the so-called Gamble’s solution mimicking the fluids found in the human lung, except for Mg and Ca salts that were replaced by Na salts to avoid interferences with the release of those structural cations occurring in the tested amphiboles. Phosphate salts were also avoided because of possible interference with Si during ICP-OES analysis. The solution pH was adjusted to pH 4.5 (i.e. that typical of macrophages) by the proper addition of HCl.

An amount of 20 mg of sample was placed in a Falcon™ polypropylene tube, suspended and then stirred in 40 mL of MGS. As a result, the suspension density^8^ of the samples of crocidolite and tremolite was of 4.33 and 1.38 m^2^/L, respectively. The tubes were fully immersed in a thermostatic water-bath and maintained at the temperature of 37 ± 1 °C for the entire duration of the experiments. A blank sample was always prepared following the same protocol for each set of experiments.

At the end of each experiment the solution was sampled with a syringe from the tube and filtered using a nitrocellulose membrane filter of 0.22 µm. Fibres recovered on filters were rinsed with ultrapure deionized water to eliminate residues of the solution and then stored under argon prior to the XPS, TEM, and XRPD measurements. The list of the samples and their labels are reported in Table 2.

**Table 2:** List and labelling of the analysed samples.

Amphibole	Incubation time				
	1 h	24 h	48 h	168 h (1 week)	720 h (1 month)
Crocidolite	C-1 h	C-24 h	C-48 h	C-1 W	C-1 M
Tremolite	T-1 h	T-24 h	T-48 h	T-1 W	T-1 M

### ICP-OES investigation

The amount of the leached cations from the fibres (Si, Mg, Ca, and Fe) was measured following the method reported in Pacella and coauthors^23^. Briefly, one cm^3^ of each filtered solution was diluted (1:20) with a 1% nitric acid solution and analysed by using a Perkin–Elmer Optima 2000 DV ICP-OES spectrometer (Perkin-Elmer, USA), equipped with a cross flow nebulizer placed inside a Scott spray chamber. Notably, it was no possible measure the Na release due to its high content in the MGS used for the experiments. ICP Aristar (BDH) standard solutions in nitric acid for the investigated elements were used to prepare the calibrating solutions for ICP-OES analyses. Data reported are the average values of triplicate measurements (corrected for the blank).

### FE-SEM investigation

SEM images were collected using a Field Emission (FE) SEM Zeiss Gemini 500. Each sample was mounted on the stub with conductive carbon tape and a thin film (5 nm) of chromium was deposited on the sample surface using a Quorum Q 150 T ES sputter to make it conductive for measurement purposes.

### HR-TEM investigation

HR-TEM investigations were carried out at the Platform of Microscopy of the University of Milano-Bicocca using a JEOL JEM 2100P instrument, operated at 200 kV. The instrument is equipped with a LaB_6_ source, a 9 Mpixel Gatan CMOS camera for image acquisition and an Oxford Energy Dispersive System (EDS) for microanalysis and can work in STEM (scanning) mode. The nominal point-to-point image resolution at 200 kV is 2.3 Å and the nominal analytical resolution at the Mn Kα (FWHM) is 124 eV.

Aliquots of the dry samples C-1 M and T-1 M were individually powdered in agate mortar, dispersed in ethanol and ultrasonicated for two minutes. The suspensions were let to set for five minutes, then an aliquot of 5 μl was pipetted on lacey carbon films supported on 300 mesh Cu-grids. The grids were carbon coated with a 4 nm film to increase conductivity, therefore, to reduce crystal warming and eventually prolong integrity during the observations.

### X-ray powder diffraction

X-ray powder diffraction (XRPD) data were collected on a Bruker AXS D8 Advance (Bruker AXS, Karlsruhe, Germany) operating in θ/θ geometry in transmission mode. The instrument is fitted with incident-beam focussing x-ray (Göbel) mirrors and a position sensitive detector VÅNTEC-1. Samples were prepared as capillaries, using 0.7 mm diameter borosilicate glass tubes. Data were measured in the 7–145° 2θ range, 0.022° 2θ step size, 8 s counting time using Cu Kα_1,2_ radiation. Structure refinements were performed by the Rietveld method using Topas V6^39^ which implements the Fundamental Parameters Approach for describing the peak shape^40^. Absorption effects for a cylindrical sample were modelled using the equation of Sabine and co-authors^41^. The procedure previously described by Ballirano and Maras^42^ was applied for handling the correlation existing between displacement parameters and absorption. Spherical harmonics were used to correct for minor preferred orientation effects, choosing the number of appropriate terms (4th-order, eight refinable parameters) according to Ballirano^43^. Input structural data for both amphiboles were those reported by Pacella and co-authors^14^. Crocidolite and tremolite were analysed both as pristine and after 720 h of incubation time (C-1 M and T-1 M, respectively).

Data of the QPA of the UICC crocidolite samples are reported in Table 1 and miscellaneous data of the refinements in Supplementary material (Table S1).

### XPS investigation

UICC crocidolite and tremolite XPS spectra were obtained using a Theta Probe angle-resolved x-ray photoelectron spectrometer (Thermo Fisher Scientific, Waltham MA, USA). The fibres were deposited on nitrocellulose membrane filters, mounted on a standard sample platen for XPS analysis. On each sample three different areas (regions) were analysed, and the data provided in the following sections are the average values with the standard deviation in parentheses. The spectra were collected using a monochromatic Al Kα_1,2_ source (hν = 1486.6 eV) selecting a 400 μm spot size. A flood gun neutralizer was used for charge compensation. All the spectra were acquired in the fixed analyser transmission (FAT) mode and the pass energy (PE) was set at 200 eV for the survey spectra, while for high resolution spectra the PE was of 100 eV. A periodic calibration following ISO 15,472:2014 was performed to verify the linearity of the binding energy scale. The binding energy scale was referenced to the adventitious aliphatic carbon component at 285.0. Further details on the spectra processing are provided in our previous works^23,24^. The composition of the fibres is given in atomic percentages and it was calculated using the first principles approach: peak intensities were corrected by the elemental sensitivity factors calculated using Scofield’s photoionization cross-sections, Reilmann’s asymmetry parameter, the transmission function correction together with the electron inelastic mean free path^24^.

## Supplementary Information

Supplementary Information.
