# Supplementary material for: Surface and bulk modifications of amphibole asbestos in mimicked gamble's solution at acidic PH
Source: Sci Rep. 2021 Jul 9;11:14249. doi: 10.1038/s41598-021-93758-9 (PMC8270982; doi:10.1038/s41598-021-93758-9)
Supplement: Supplementary file 1 — Supplementary Information. [file 41598_2021_93758_MOESM1_ESM.pdf]

REVISION 1

SURFACE AND BULK MODIFICATIONS OF AMPHIBOLE ASBESTOS IN MIMICKED GAMBLE'S SOLUTION AT ACIDIC PH

Alessandro Pacella[1*], Paolo Ballirano[1], Marzia Fantauzzi[2], Antonella Rossi[2], Elisa Nardi[3], Giancarlo Capitani[4], Lorenzo Arrizza[5], and Maria Rita Montereali[6]

[1] Dipartimento di Scienze della Terra and Laboratorio Rettorale Fibre e Particolato Inorganico, Sapienza Università di Roma, P.le A. Moro 5, I-00185, Rome, Italy

[2] Dipartimento di Scienze Chimiche e Geologiche, INSTM research unit, Centro Grandi Strumenti Università di Cagliari, I-09042 Monserrato, Cagliari, Italy

[3] Istituto Superiore per la Protezione e la Ricerca Ambientale (ISPRA), via Vitaliano Brancati 48, 00144 Roma, Italy

[4] Dipartimento di Scienze dell'Ambiente e di Scienze della Terra, Università degli Studi di Milano-Bicocca, Piazza della Scienza 4, 20126, Milano, Italy

[5] Centro di Microscopie, Università degli Studi dell'Aquila, Via Vetoio (Coppito 1, Edificio "Renato Ricamo"), 67100, Coppito, L'Aquila, Italy

[6] ENEA, C.R. Casaccia via Anguillarese 301, I-00123 S. Maria di Galeria, Roma, Italy

* Corresponding authors: alessandro.pacella@uniroma1.it

Table S1. Cell parameters and volume of UICC crocidolite fibres and agreement factors (as defined in the work of Young[44]) of the Rietveld refinements.

	Pristine	C-1M
R_{Bragg} (%)	0.24	0.39
R_{wp} (%)	0.88	1.23
R_p (%)	0.59	0.83
GoF	2.38	2.73
DWd	1.45	1.32
a (Å)	9.73789(14)	9.73825(14)
b (Å)	18.0504(3)	18.0512(2)
c (Å)	5.33022(7)	5.33044(6)
β (°)	103.5188(11)	103.5149(10)
Vol. (Å^3)	910.95(2)	911.08(2)

Table S2. Relevant bond distances (in Å) of UICC crocidolite fibres. * Calculated as in Table 7 in the work of Hawthorne and Oberti[45].

		Pristine	C-1M
T(1)	-O(7)	1.617(5)	1.612(4)
	-O(6)	1.649(7)	1.630(7)
	-O(1)	1.621(10)	1.612(10)
	-O(5)	1.607(9)	1.620(9)
<*T*(1)-O>		1.624	1.619
T(2)	-O(4)	1.575(8)	1.578(8)
	-O(5)	1.653(8)	1.654(8)
	-O(2)	1.629(10)	1.627(10)
	-O(6)	1.662(8)	1.664(8)
<*T*(2)-O>		1.630	1.631
M(1)	-O(3) x2	2.110(7)	2.111(7)
	-O(1) x2	2.097(9)	2.116(9)
	-O(2) x2	2.116(8)	2.124(7)
<*M*(1)-O>		2.108	2.117
M(2)	-O(4) x2	1.941(7)	1.950(7)
	-O(2) x2	2.059(9)	2.054(9)
	-O(1) x2	2.134(8)	2.124(8)
<*M*(2)-O>		2.045	2.043
M(3)	-O(1) x4	2.139(8)	2.141(8)
	-O(3) x2	2.123(13)	2.129(12)
<*M*(3)-O>		2.134	2.137
<<*M*(1,2,3)-O>>		2.095	2.099
<<$r^{M(1,2,3)}$>>*		0.743	0.748
M(4)	-O(4) x2	2.332(9)	2.328(9)
	-O(2) x2	2.409(9)	2.414(8)
	-O(6) x2	2.506(8)	2.484(8)
	-O(5) x2	2.859(8)	2.858(7)
<*M*(4)-O>		2.527	2.521

Table S3. Site scattering (*s.s.*) at A, B and C sites from Rietveld refinement.

Site	Pristine	C-1M
C		
M(1)	44.8(2)	44.3(2)
M(2)	50.1(3)	49.5(3)
M(3)	23.73(16)	24.12(15)
**$\Sigma_{M(1)+M(2)+M(3)}$**	**118.6(7)**	**117.9(7)**
B		
M(4)	21.91(19)	21.93(19)
A	2.02(11)	1.88(11)

Table S4. Site partition at A, B and C sites from Rietveld refinement.

	Fe^{2+}/Fe^{3+} partition from <r^M>	
Site	Pristine	C-1M
C		
M(1)	$Mg_{0.51(2)}Fe^{3+}_{0.28}Fe^{2+}_{1.21}$	$Mg_{0.55(2)}Fe^{3+}_{0.11}Fe^{2+}_{1.34}$
M(2)	$Mg_{0.14(2)}Fe^{3+}_{1.34}Fe^{2+}_{0.52}$	$Mg_{0.18(2)}Fe^{3+}_{1.34}Fe^{2+}_{0.48}$
M(3)	$Mg_{0.16(1)}Fe^{3+}_{0.00}Fe^{2+}_{0.84}$	$Mg_{0.13(1)}Fe^{3+}_{0.00}Fe^{2+}_{0.87}$
**$\Sigma_{M(1)+M(2)+M(3)}$**	**$Mg_{0.81(5)}Fe^{3+}_{1.62}Fe^{2+}_{2.57}$**	**$Mg_{0.86(5)}Fe^{3+}_{1.49}Fe^{2+}_{2.69}$**
Fe^{2+}/Fe^{3+}	1.59	1.81
B		
M(4)	$Na_{1.991(17)}$	$Na_{1.993(17)}$
A(2/*m*)	$Na_{0.184(10)}$	$Na_{0.171(10)}$

Table S5. Results of ICP-OES analyses of UICC crocidolite fibres after incubation in the mimicked Gamble's solution at pH 4.5 for 1, 24, 48, 168 and 720 hours. Standard deviations (in parentheses) were calculated over three independent measurements.

Sample	Fe (mg/kg)	Ca (mg/kg)	Mg (mg/kg)	Si (mg/kg)
C-1h		10814(895)	586(39)	221(37)
C-24h	1205(71)	11085(2299)	786(167)	1890(288)
C-48h	2216(368)	11897(288)	1168(44)	4436(314)
C-1W	2563(386)	8148(727)	1383(148)	6019(994)
C-1M		10148(366)	2307(163)	10900(1031)

Table S6. Binding energy values (eV) of the main photoelectron lines in UICC crocidolite samples. Average values and standard deviation (in parentheses) over three measurements carried out on different areas of the same sample.

		Binding Energy (eV)				
		**C-1h**	**C-24h**	**C48-h**	**C-1W**	**C-1M**
**Si 2p$_{3/2}$**		102.5(0.1)	102.4(0.1)	102.4(0.2)	102.5(0.1)	102.4(0.1)
**O 1s**	O^{2-}	530.1(0.2)	530.1(0.2)	530.1(0.2)	530.2(0.2)	530.1(0.2)
	NB-O† and OH^-	531.2(0.2)	531.1(0.2)	531.1(0.2)	531.1(0.2)	531.1(0.2)
	B-O‡	532.2(0.2)	532.1(0.2)	532.1(0.2)	532.2(0.2)	532.1(0.2)
**Fe 2p$_{3/2}$**	Fe (II) - O	709.1(0.2)	709.2(0.2)	709.1(0.2)	709.3(0.2)	709.2(0.2)
	Fe (III) - O	710.5(0.2)	710.6(0.2)	710.6(0.2)	710.8(0.2)	710.6(0.2)
	FeOOH	711.7(0.1)	711.6(0.1)	711.7(0.1)	711.8(0.1)	711.6(0.1)
**Na 1s**		1072.2(0.2)	1072.1(0.1)	1072.1(0.1)	1072.1(0.2)	1072.1(0.1)
**Mg 2p**		49.4(0.1)	49.4(0.1)	49.4(0.1)	49.4(0.1)	49.4(0.1)

† NB-O: non-bridging oxygen in silicates. ‡ B-O: bridging oxygen in silicates.

Table S7. Surface quantitative composition (at.%) of both pristine and incubated UICC crocidolite samples for 1h (C-1h), 24 hours (C-24h), 48 hours (C-48h), 163 hours (C-1W) and 720 hours (C-1M) in the mimicked Gamble's solution. Average values and standard deviation (in parentheses) over three measurements carried out on different areas of the same sample.

Sample	Oxygen (at.%)	Silicon (at.%)	Iron (at.%)	Sodium (at.%)	Magnesium (at.%)
Pristine	65.0(0.3)	23.5(0.8)	5.0(0.2)	3.5(0.7)	3.0(0.2)
C-1h	63(2)	28(2)	5.3(0.4)	3.4(0.3)	0.4(0.1)
C-24h	63.9(0.6)	25.8(0.7)	6.2(0.3)	3.7(0.1)	0.4(0.1)
C-48h	65.0 (0.5)	24.2(0.4)	6.5(0.2)	3.8(0.2)	0.4(0.1)
C-1W	64.4(0.2)	24.6(0.3)	7.0 (0.2)	3.8(0.1)	0.33(0.01)
C-1M	65(1)	23.6(0.7)	6.9 (0.2)	4.4(0.3)	0.4 (0.1)

Table S8. Relative intensities of Fe $2p_{3/2}$ components (area%) in UICC crocidolite samples.

Sample	Fe(II)-O (area%)	Fe(III)-O (area%)	Fe-OOH (area%)
Pristine	21(1)	19(1)	60(1)
C-1h	24(1)	20.8(0.2)	55.3(0.2)
C-24h	25(1)	23(1)	53(2)
C-48h	30.5(0.2)	20.6(0.9)	49(1)
C-1W	28.9(0.1)	23(3)	48(3)
C-1M	29(2)	18(1)	53(1)

Table S9. Cell parameters and volume of Maryland tremolite fibres and agreement factors[53] of the Rietveld refinements.

	Pristine	T-1M
R_{Bragg} (%)	1.39	1.12
R_{wp} (%)	2.94	2.19
R_p (%)	2.21	1.64
GoF	3.96	2.71
DWd	0.52	0.75
a (Å)	9.85470(7)	9.85372(5)
b (Å)	18.08013(10)	18.07917(9)
c (Å)	5.28250(2)	5.28221(2)
β (°)	104.7494(5)	104.7506(4)
Vol. (Å^3)	910.192(9)	909.997(8)

Table S10. Relevant bond distances (in Å) of Maryland tremolite fibres. * Calculated as in Table 7 of [54].

		Pristine	T-1M
T(1)	-O(7)	1.622(2)	1.6199(19)
	-O(6)	1.654(3)	1.649(3)
	-O(1)	1.585(4)	1.588(3)
	-O(5)	1.656(4)	1.648(3)
<*T*(1)-O>		1.629	1.626
T(2)	-O(4)	1.587(3)	1.591(3)
	-O(5)	1.648(3)	1.649(3)
	-O(2)	1.628(4)	1.631(3)
	-O(6)	1.676(4)	1.675(3)
<*T*(2)-O>		1.635	1.637
M(1)	-O(3) x2	2.093(3)	2.085(2)
	-O(1) x2	2.060(3)	2.066(3)
	-O(2) x2	2.094(3)	2.098(3)
<*M*(1)-O>		2.082	2.083
M(2)	-O(4) x2	2.019(3)	2.018(3)
	-O(2) x2	2.084(3)	2.086(3)
	-O(1) x2	2.136(3)	2.132(3)
<*M*(2)-O>		2.080	2.079
M(3)	-O(1) x4	2.100(3)	2.093(2)
	-O(3) x2	2.054(4)	2.060(4)
<*M*(3)-O>		2.085	2.082
<<*M*(1,2,3)-O>>		2.082	2.081
<<$r^{M(1,2,3)}$>>*		0.727	0.726
M(4)	-O(4) x2	2.338(3)	2.332(3)
	-O(2) x2	2.398(3)	2.389(3)
	-O(6) x2	2.544(3)	2.543(3)
	-O(5) x2	2.761(3)	2.764(3)
<*M*(4)-O>		2.510	2.507

Table S11. Site scattering (*s.s.*) at A, B and C sites from Rietveld refinement.

Site	Pristine	T-1M
C		
M(1)	25.87(7)	25.97(7)
M(2)	25.16(7)	25.36(7)
M(3)	13.20(5)	13.26(5)
**$\Sigma_{M(1)+M(2)+M(3)}$**	**64.22(19)**	**64.6(2)**
B		
M(4)	39.80(8)	39.96(8)
A	1.14(5)	0.74(5)

Table S12. Site partition at A, B and C sites from Rietveld refinement for Maryland Tremolite samples.

	Fe^{2+}/Fe^{3+} partition from <r^M>	
Site	Pristine	T-1M
C		
M(1)	$[Mg_{1.87(1)}Fe^{3+}_{0.01}Fe^{2+}_{0.12}]$	$[Mg_{1.86(1)}Fe^{3+}_{0.01}Fe^{2+}_{0.13}]$
M(2)	$[Mg_{1.92(1)}Fe^{3+}_{0.03}Fe^{2+}_{0.05}]$	$[Mg_{1.90(1)}Fe^{3+}_{0.08}Fe^{2+}_{0.02}]$
M(3)	$[Mg_{0.91(1)}Fe^{3+}_{0.03}Fe^{2+}_{0.06}]$	$[Mg_{0.91(1)}Fe^{3+}_{0.02}Fe^{2+}_{0.07}]$
**$\Sigma_{M(1)+M(2)+M(3)}$**	**$[Mg_{4.70(3)}Fe^{3+}_{0.07}Fe^{2+}_{0.23}]$**	**$[Mg_{4.67(3)}Fe^{3+}_{0.11}Fe^{2+}_{0.22}]$**
Fe^{2+}/Fe^{3+}	3.43	2.00
B		
M(4)	$Ca_{1.990(4)}$	$Ca_{1.998(4)}$
A(2/*m*)	$Na_{0.103(5)}$	$Na_{0.068(5)}$

Table S13. Results of ICP-OES analyses of Maryland tremolite samples after incubation in the mimicked Gamble’s solution at pH 4.5 for 1, 24, 48, 168 and 720 hours. Standard deviations (in parentheses) were calculated over three independent measurements.

**Sample**	**Ca (mg/kg)**	**Mg (mg/kg)**	**Si (mg/kg)**
T-1h	117 (7)	146 (8)	15 (10)
T-24h	162 (25)	196 (39)	43 (1)
T-48h	161 (3)	240 (3)	88 (12)
T-1W	261 (32)	305 (33)	231 (9)
T-1M	256 (26)	373 (10)	543 (10)

Table S14. Binding energy values (eV) of the main photoelectron lines in Maryland tremolite samples. Standard deviations are in parentheses.

Binding Energy (eV)						
		**T-1h**	**T-24h**	**T-48h**	**T-1W**	**T-1M**
**Si $2p_{3/2}$**		102.2(0.1)	102.1(0.1)	102.4(0.2)	102.3(0.1)	102.1(0.1)
**O 1s**	O^{2-}	529.0(0.2)	530.2(0.2)	530.1(0.2)	530.3(0.2)	530.2(0.2)
	NB-O† and OH^-	531.1(0.2)	531.2(0.2)	531.2(0.2)	531.3(0.2)	531.2(0.2)
	B-O‡	532.2(0.2)	532.5(0.2)	532.1(0.2)	532.5(0.2)	532.1(0.2)
**Fe $2p_{3/2}$**	Fe (II) - O	708.9(0.2)	709.2(0.2)	709.1(0.2)	709.1(0.2)	709.1(0.2)
	Fe (III) - O	710.5(0.2)	710.6(0.2)	710.5(0.2)	710.8(0.2)	710.8(0.2)
	FeOOH	711.5(0.1)	711.7(0.2)	711.6(0.2)	711.6(0.1)	711.8(0.2)
**Na 1s**		1072.2(0.2)	1071.9(0.1)	1072.1(0.1)	1071.8(0.2)	1072.1(0.1)
**Mg 2p**		49.2(0.1)	49.4(0.1)	49.4(0.1)	49.5(0.1)	49.5(0.1)
**Ca 2p**		347.7(0.2)	347.6(0.2)	347.6(0.2)	347.4(0.1)	347.4(0.2)

† NB-O: no bridging oxygen in silicates
‡ B-O: bridging oxygen in silicates

Table S15. Surface quantitative composition at% of Maryland tremolite samples incubated for 1hour (T-1h), 24 hours (T-24h), 48 hours (T-48h), one week (T-1W) and one month (T-1M) in the mimicked Gamble's solution. Average values and standard deviation (in parentheses) over three measurements**.**

**Sample**	**Oxygen (at%)**	**Silicon (at%)**	**Iron (at%)**	**Sodium (at%)**	**Magnesium (at%)**	**Calcium (at%)**
Pristine	61.4(0.3)	27.1(0.3)	1.5(0.1)	-	6(1)	4.0(0.2)
T-1h	61(2)	31(3)	1.3(0.1)	1.0(0.2)	2.7(0.4)	3.2(0.2)
T-24h	64.1(0.1)	26.2(0.3)	1.4 (0.1)	1.2(0.1)	3.1(0.1)	4.0(0.2)
T-48h	64.5(0.1)	25.4(0.1)	1.4(0.1)	1.1(0.1)	3.3(0.1)	4.4(0.1)
T-1W	63(2)	28(3)	1.3(0.1)	0.6(0.3)	3.0(0.3)	3.8(0.5)
T-1M	64.0(0.7)	27(1)	1.3(0.1)	0.8(0.1)	3.1(0.1)	4.2(0.4)

Table S16. Relative intensities of Fe $2p_{3/2}$ components (area %) in Maryland tremolite samples.

**Sample**	**Fe(II)-O (area%)**	**Fe(III)-O (area%)**	**Fe-OOH (area%)**
Pristine	29	10	61
T-1h	11(2)	5(1)	84(3)
T-24h	13(1)	2(1)	85(1)
T-48h	19(1)	3(1)	78(2)
T-1W	13(1)	10(2)	77(3)
T-1M	15(2)	8(2)	77(2)

Figures

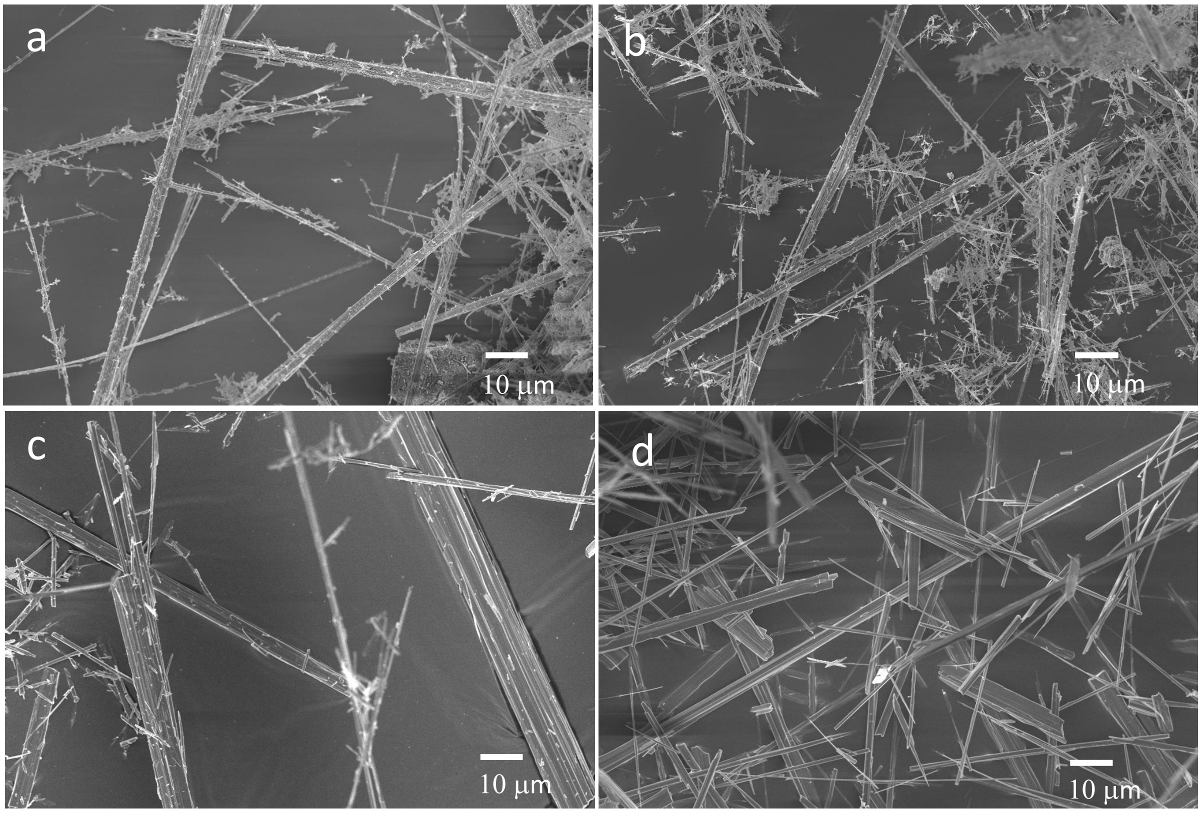

Figure S1. FE-SEM images of the investigates fibrous samples: a) pristine UICC crocidolite, b) treated UICC crocidolite fibres (C-1M); c) pristine Maryland tremolite fibres, d) treated Maryland tremolite fibres (T-1M).

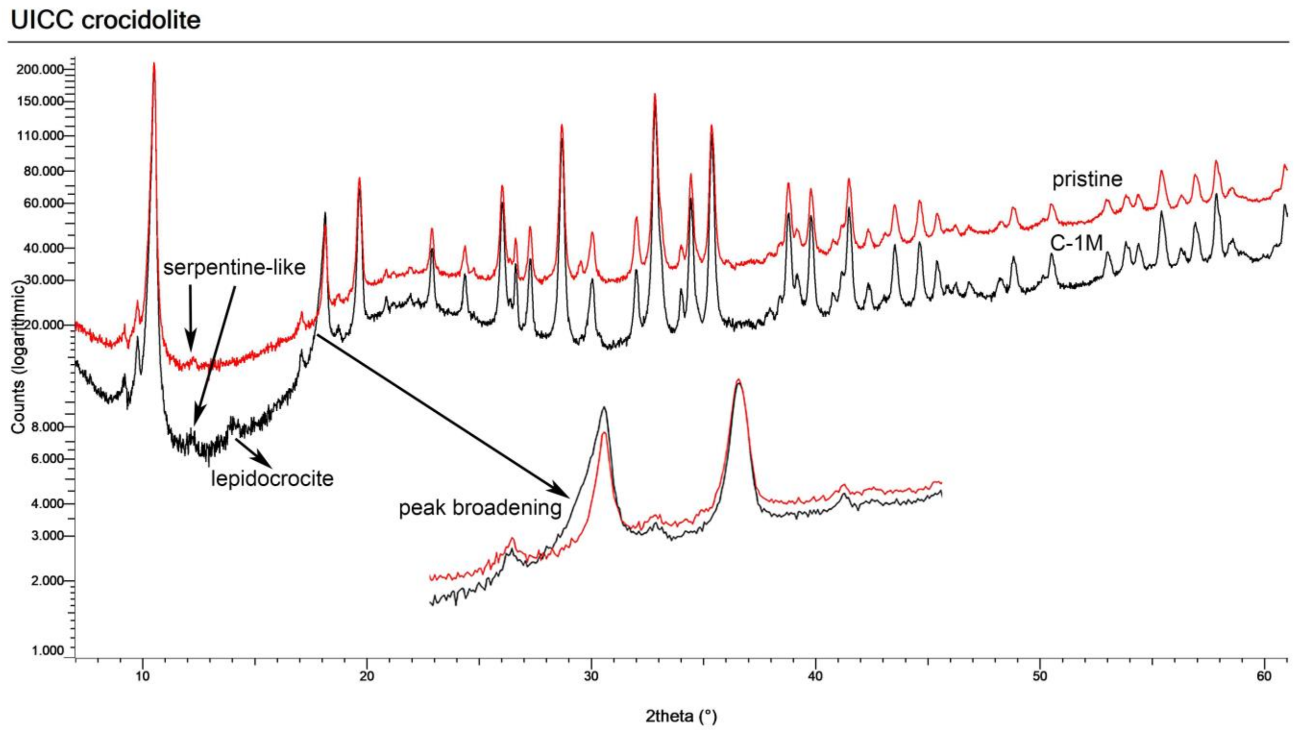

Figure S2. Experimental XRPD data of UICC crocidolite samples. Pristine sample (red) has been vertically displaced for clarity. The 002 reflection of the serpentine-like phase and the 200 reflection of lepidocrocite are arrowed. Inset: magnified view of the $11\bar{1}$ reflection of crocidolite (arrowed), lying at ca. 18° 2θ, showing its marked broadening toward lower angles upon prolonged incubation in mimicked Gamble's solution due to the occurrence of the very broad most intense reflection common to several hydrated sulphates of Mg and Fe.

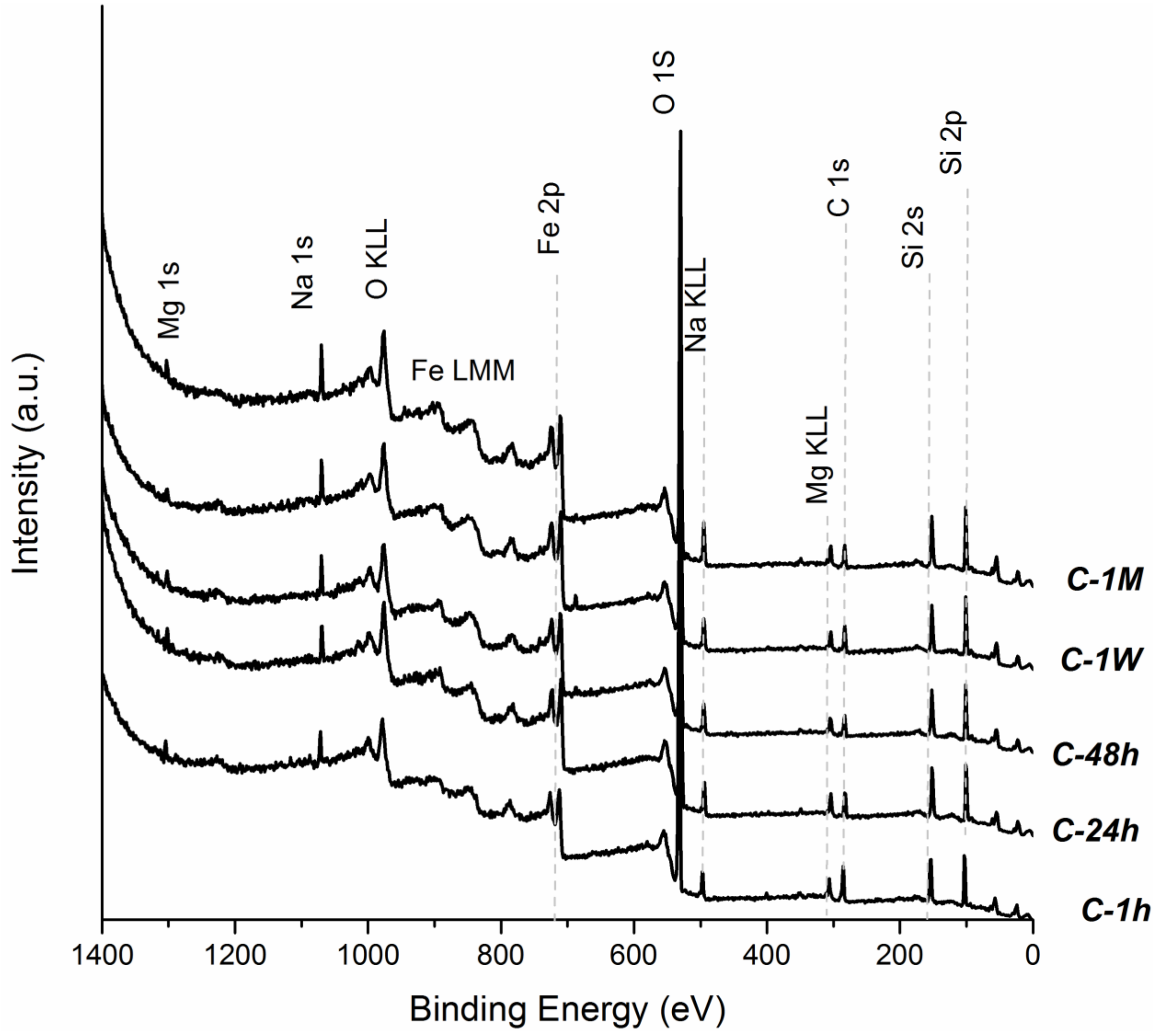

Figure S3. Survey spectra of UICC crocidolite samples. X-ray source: Al Kα.

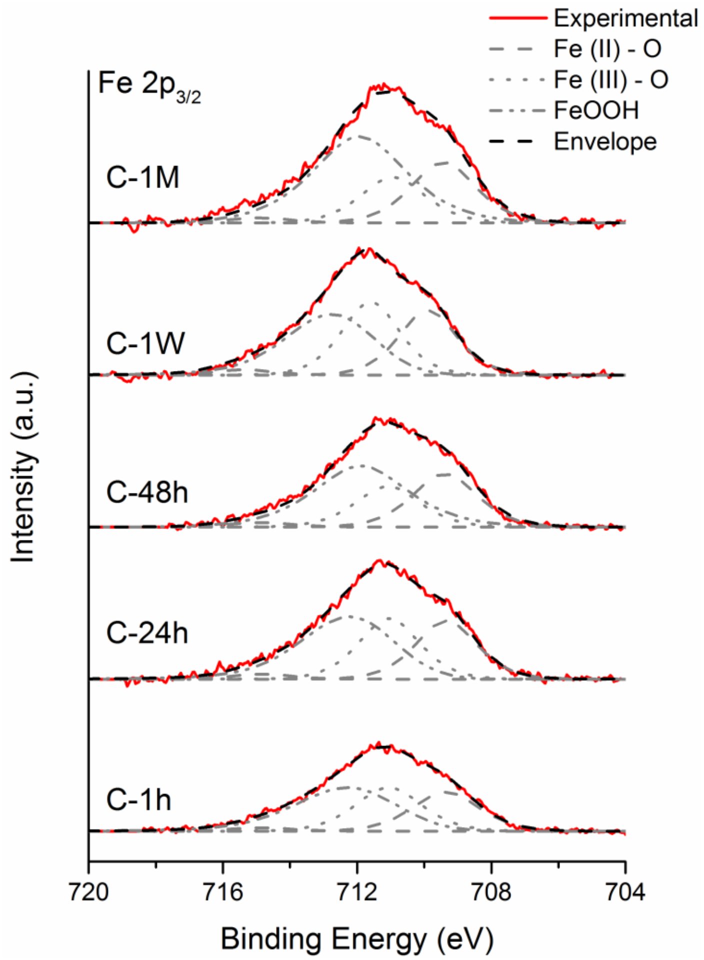

Figure S4. Fe $2p_{3/2}$ peaks of the UICC crocidolite samples incubated for 1h (C-1h), 24 h (C-24h), 48 h (C-48h), 168 h (C-1W) and 720 h (C-1M).

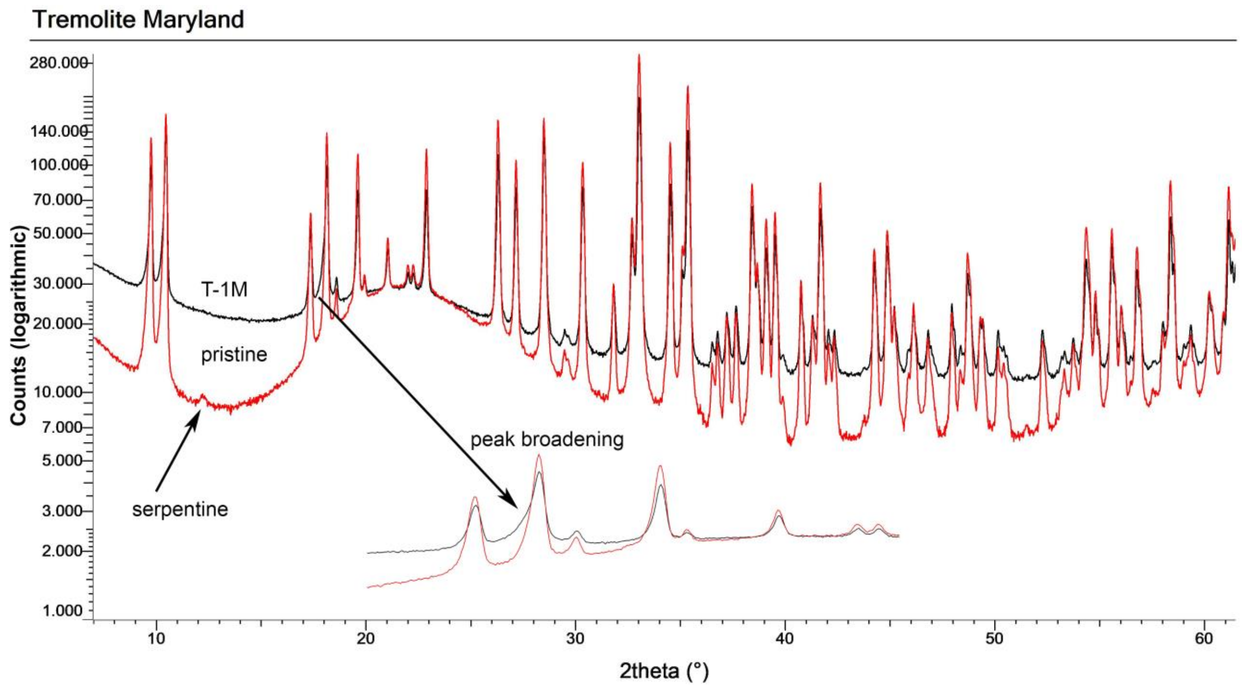

Figure S5. Experimental XRPD data of tremolite from tremolite Maryland samples. Pristine sample (red) has been vertically displaced for clarity. The 002 reflection of serpentine is arrowed. Inset: magnified view of the $11\bar{1}$ reflection of tremolite (arrowed), lying at ca. 18° 2θ, showing its marked broadening toward lower angles upon prolonged incubation in simplified Gamble's solution due to the occurrence of the very broad most intense reflection common to several hydrated sulphates of Mg and Fe.

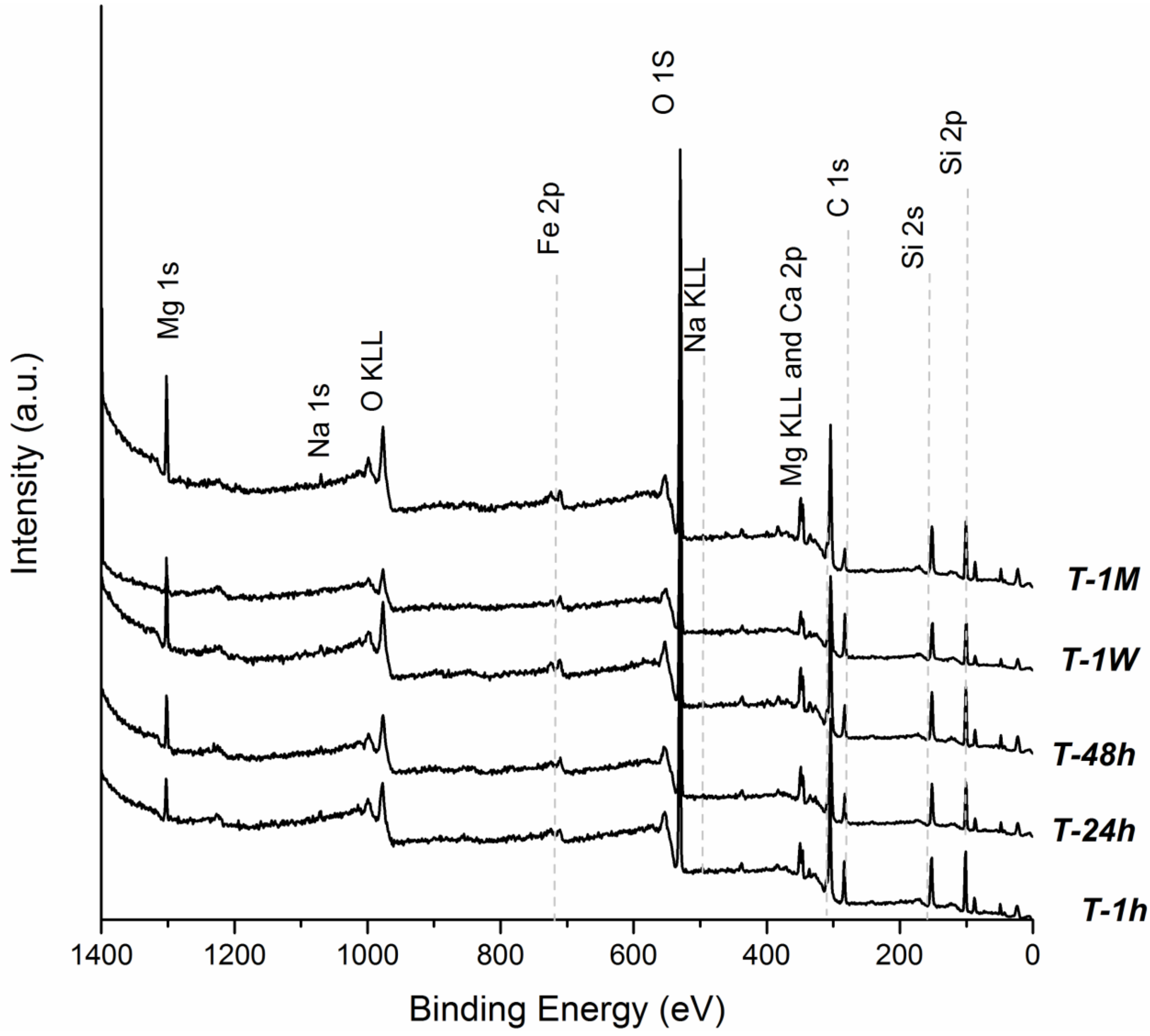

Figure S6. Survey spectra of tremolite Maryland samples. X-ray source: Al Kα.

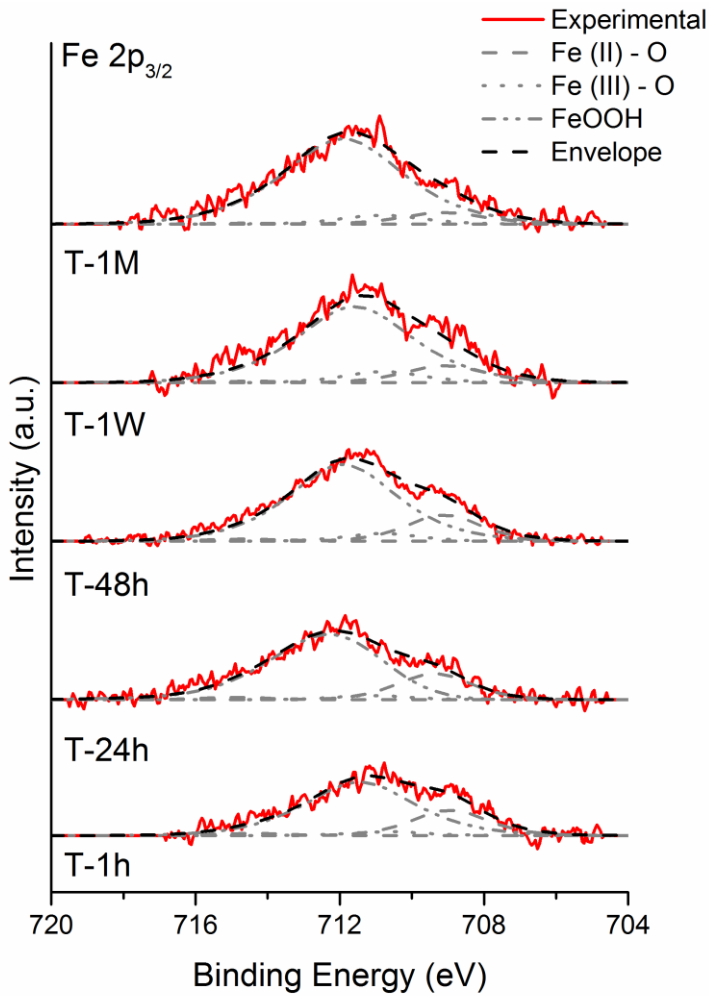

Figure S7. Fe $2p_{3/2}$ peaks of the tremolite Maryland samples incubated for 1hour (T-1h), 24 hours (T-24h), 48 hours (T-48h), 168 hours (T-1W) and 720 hours (T-1M).
